# Supplementary material for: Boolean logic algebra driven similarity measure for text based applications
Source: PeerJ Comput Sci. 2021 Jul 29;7:e641. doi: 10.7717/peerj-cs.641 (PMC8330432; doi:10.7717/peerj-cs.641)
Supplement: Supplemental Information 1 [file peerj-cs-07-641-s001.pdf]

Appendix (sample for review)

In the next pages, we provide only a sample of the averaged results over the whole number of features on both datasets for BLAB-SM and SMTP similarity measures. The ultimate purpose of this sample is to bring the kind attention of the respected Editorial Board and the prospective reviewers into the full picture of the work. The code (that produces all results) is provided on GitHub via this link https://github.com/aliamer/Boolean-Logic-Algebra-Driven-Similarity-Measure-for-Text-Based-Applications

Moreover, a sample of results of jaccard is provided to prove that jaccard is a poor choice when dealing with tfidf based document matching.

BLAB-SM Similarity Measure

No. of features	Metric	Measure	k	Reuters-8	Web-Kb
ALL	BLAB_SM	Accuracy	1	0.9302426343154246	0.7777777777777778
			3	0.932842287694974	0.7888888888888889
			5	0.9376083188908145	0.8206349206349206
			7	0.9345753899480069	0.8293650793650794
			9	0.9332755632582322	0.8238095238095238
			11	0.9324090121317158	0.8214285714285714
			13	0.9280762564991335	0.8182539682539682
			15	0.9233102253032929	0.8158730158730159
			17	0.9194107452339688	0.8214285714285714
			19	0.9211438474870017	0.8246031746031746
			21	0.9150779896013865	0.8238095238095238
			23	0.9172443674176777	0.8269841269841269
			25	0.9168110918544194	0.8214285714285714
			27	0.9137781629116117	0.8277777777777777
			29	0.912478336221837	0.8214285714285714
			31	0.9120450606585788	0.819047619047619
			33	0.9133448873483535	0.8166666666666667
			35	0.9103119584055459	0.8182539682539682
			37	0.9077123050259965	0.8182539682539682
			39	0.9081455805892548	0.8222222222222222
			41	0.9055459272097054	0.8166666666666667
			43	0.9072790294627383	0.8142857142857143
			45	0.9055459272097054	0.8134920634920635
			47	0.9042461005199307	0.8158730158730159
			49	0.9038128249566725	0.8134920634920635
			51	0.9055459272097054	0.8134920634920635
			53	0.9033795493934142	0.8158730158730159
			55	0.9020797227036396	0.8182539682539682
			57	0.9016464471403813	0.8150793650793651
			59	0.9003466204506065	0.8166666666666667

			61	0.901213171577123	0.8174603174603174
			63	0.8977469670710572	0.8142857142857143
			65	0.8977469670710572	0.8150793650793651
			67	0.8977469670710572	0.8103174603174603
			69	0.8960138648180243	0.8142857142857143
			71	0.8964471403812825	0.8134920634920635
			73	0.8947140381282496	0.8119047619047619
			75	0.8947140381282496	0.8126984126984127
			77	0.8929809358752167	0.8103174603174603
			79	0.8921143847487002	0.8111111111111111
			81	0.8912478336221837	0.8103174603174603
			83	0.8903812824956673	0.8087301587301587
			85	0.8903812824956673	0.8095238095238095
			87	0.8890814558058926	0.8103174603174603
			89	0.8895147313691508	0.8142857142857143
			91	0.8890814558058926	0.807936507936508
			93	0.8882149046793761	0.8047619047619048
			95	0.8886481802426344	0.8071428571428572
			97	0.8882149046793761	0.8047619047619048
			99	0.8873483535528596	0.8055555555555556
			101	0.8864818024263431	0.8047619047619048
			103	0.8860485268630849	0.803968253968254
			105	0.8851819757365684	0.8023809523809524
			107	0.8834488734835355	0.8007936507936508
			109	0.8830155979202773	0.8007936507936508
			111	0.8830155979202773	0.7992063492063493
			113	0.8804159445407279	0.8007936507936508
			115	0.8804159445407279	0.8007936507936508
			117	0.8791161178509532	0.7984126984126985
			119	0.8782495667244368	0.7984126984126985
			Average	0.9023035817446563	0.812261904761905

No. of features	Metric	Measure	k	Reuters-8	Web-Kb
ALL	BLAB_SM	Precision	1	0.8854672928291207	0.763044697748645
			3	0.8897512106788055	0.8289770442776742
			5	0.9040055474785454	0.8569434576487417
			7	0.8968417008201113	0.8740917229792855
			9	0.9029308192561671	0.8698667791655103
			11	0.9119939660167289	0.8687958023266296
			13	0.8931815409653767	0.8658929595689282
			15	0.886874335698371	0.866190740275685
			17	0.8745262402630039	0.8703484113002906
			19	0.8839450687079726	0.8725564962706431
			21	0.8720880771049108	0.8720743573739801

			23	0.8760182937940711	0.8754934091116691
			25	0.8704208837664332	0.8716148303667356
			27	0.8672355524117455	0.8751152166510207
			29	0.8668301562289489	0.8716299140151688
			31	0.8593712776356528	0.8695109227760474
			33	0.8582159399744141	0.8683853142298346
			35	0.8702989438121966	0.8696988405298413
			37	0.8671810759936112	0.86932283655253
			39	0.8993261189375525	0.8714360373809589
			41	0.896454966586703	0.8681212567453549
			43	0.8983228483761032	0.8662763441447421
			45	0.8986654682002241	0.8661494340574037
			47	0.8959984931407865	0.8681073245604715
			49	0.8983345972666194	0.8660955518791049
			51	0.8954951505861202	0.8657740714739761
			53	0.8953749532087146	0.8681440162271805
			55	0.8936806214329377	0.8688811417206208
			57	0.8928109783930268	0.867297551685776
			59	0.8921595434103977	0.8696002450192062
			61	0.8926362002020128	0.8701024122915328
			63	0.8900632765090578	0.8667825559142421
			65	0.8898224088229278	0.8674428408901416
			67	0.8907351842238409	0.8636666248695113
			69	0.8892131655984723	0.8677689585355133
			71	0.8941049435355359	0.8670553477801377
			73	0.8848248337763078	0.8668435753748139
			75	0.8863126236538215	0.8658075895214936
			77	0.883240545695896	0.8627243918206033
			79	0.882530812504329	0.8639379829004734
			81	0.8815511192717329	0.863798783452685
			83	0.8823070889172527	0.8627665984261121
			85	0.7582509817280293	0.8631217933160306
			87	0.7565362336937878	0.8635557871668762
			89	0.7599501469007665	0.8664846579973577
			91	0.759500067980406	0.8612413628422708
			93	0.755662098458009	0.8591880513365837
			95	0.881681464689509	0.8621705921958873
			97	0.8821085315276285	0.859465129925488
			99	0.7541643551898514	0.8605128257640084
			101	0.752042859690683	0.8602314556816806
			103	0.7498655565387127	0.8591887761679045
			105	0.7437788816301982	0.8582046566285491
			107	0.7473290124181355	0.8573675995007649
			109	0.7479752993375706	0.856779306609677
			111	0.747986124132421	0.8673161041071942
			113	0.743297211982727 5	0.8689685083496245

			115	0.7423716729958669	0.8693227392845355
			117	0.7423474239455703	0.8673866337292298
			119	0.7405244877076635	0.8675771397802151
			Average	0.8501086046039016	0.8640369585037467

**No. of features**	**Metric**	**Measure**	**k**	**Reuters-8**	**Web-Kb**
ALL	BLAB_SM	Recall	1	0.8364233153003058	0.7239316057848948
			3	0.8171698668800876	0.7203487878101436
			5	0.8323036925473892	0.751834479568155
			7	0.8078475119429838	0.7628349519933117
			9	0.7998599074359378	0.7545369196775191
			11	0.781502589559804	0.7497059057243831
			13	0.7716677326967578	0.7475232905734012
			15	0.7627624336092724	0.7424575362491347
			17	0.7390772702748225	0.7466309303168198
			19	0.7498930083292341	0.7488135454678017
			21	0.7248122128804134	0.7457746462100062
			23	0.7264469508056655	0.7481736576111422
			25	0.7185219362876631	0.7397214077102907
			27	0.7102950279828326	0.7434144169940076
			29	0.7053853845378748	0.7398019304963622
			31	0.705692987470506	0.7335322957464927
			33	0.7012622717501167	0.7330606604125945
			35	0.7036574020435369	0.7301849613185791
			37	0.6938974013399638	0.7282768597795845
			39	0.692267316623974	0.7321956268557108
			41	0.6806696202818115	0.7252556925505802
			43	0.6878568150827724	0.7220426890872975
			45	0.6736710833389024	0.7186331759942889
			47	0.6708767090868857	0.7208403511611462
			49	0.6671933640423742	0.7169414707054482
			51	0.6780453466642533	0.7164166392851762
			53	0.6726869146248307	0.7197101655345306
			55	0.6607032503994579	0.7205524147036417
			57	0.6595802709508045	0.7189683257719194
			59	0.6579482966737816	0.7237182048467986
			61	0.6594740687417986	0.724404693931399
			63	0.6529612000909624	0.7196097561280308
			65	0.6513983093682818	0.7187335854007888
			67	0.6476840148979287	0.7115411786957364
			69	0.6429647596543226	0.7189860578007107
			71	0.6472830164133232	0.7163361164991047
			73	0.6372370170152657	0.714698219388823
			75	0.6368157708334283	0.7149152277311623

			77	0.6290060586643664	0.7095593823577542
			79	0.6253612140986331	0.7112955342828987
			81	0.6148179851791583	0.7100842137780263
			83	0.6135957115415809	0.7091505377811365
			85	0.6051197003774327	0.7094228968014866
			87	0.6013154569058505	0.7090162068165058
			89	0.602839847149753	0.713089191477691
			91	0.600326453133172	0.7047283684046183
			93	0.5984400726203608	0.7019957497253038
			95	0.6047521044341463	0.7065736791863326
			97	0.6022534465052913	0.7013720513979836
			99	0.5911578391155561	0.7029471577509849
			101	0.588364846687654	0.7021316231893863
			103	0.5880783402562508	0.7000870360941238
			105	0.5827265453129076	0.6992270548962214
			107	0.581335161231509	0.6975891577859399
			109	0.5791321736654494	0.6969101087806089
			111	0.5787029894032316	0.6955069520414578
			113	0.5732389981876576	0.6963669332393603
			115	0.5723800966431035	0.6961321928682298
			117	0.5667687502047436	0.6944566771087286
			119	0.5637555712515516	0.6942219367375981
			Average	0.665487756850495	0.720448717066988

**No. of features**	**Metric**	**Measure**	**k**	**Reuters-8**	**Web-Kb**
ALL	BLAB_SM	F Measure	1	0.8580264098679282	0.7342212535380959
			3	0.8467937587288485	0.7375615144377822
			5	0.8621846295200323	0.7689605449028104
			7	0.8414097775794236	0.7814391182747872
			9	0.8385343239839316	0.770989207406206
			11	0.8252765778918649	0.7658918834809404
			13	0.8138129384179085	0.7632634866185875
			15	0.8060756605865587	0.7587953091785058
			17	0.7825489714526759	0.7630381607162916
			19	0.7959809676883773	0.7654063459138897
			21	0.7721178617803538	0.7615238773853423
			23	0.7743820227954321	0.7653456917661463
			25	0.763289596023708	0.7546115444390523
			27	0.7561110796855177	0.7569608768473808
			29	0.750976583104018	0.7545370977450918
			31	0.7501708426026874	0.7455712927301495
			33	0.7443960038434292	0.7471161224494136
			35	0.7533623830872845	0.7413079020573935
			37	0.7452867016402287	0.7376615263723527

			39	0.7455998638864398	0.7420433700961337
			41	0.733887084798955	0.7342069762908683
			43	0.7418320203202213	0.7302771019372912
			45	0.7246258659143779	0.7244295393371144
			47	0.7221118990230886	0.72776886803631
			49	0.71985142761094	0.7221447538419343
			51	0.7348868558436732	0.7203579893935869
			53	0.7304071046527277	0.7246008764098726
			55	0.7150115845349121	0.7240253353019769
			57	0.7138394703194568	0.7237842648092081
			59	0.7126818472243981	0.7328094135573204
			61	0.7137033146720118	0.732083517804328
			63	0.7085708050111357	0.7253510865735322
			65	0.7061811590415745	0.7239050919211715
			67	0.7028592435676722	0.7141781390428374
			69	0.697927118097579	0.7254380416244841
			71	0.704152567205142	0.7210564736071559
			73	0.6911158606405507	0.7199957283567127
			75	0.6916590725477058	0.7182439110706116
			77	0.6823578583351153	0.7097694290987795
			79	0.6801979123956414	0.7125025638482325
			81	0.664436470664489	0.7119066662567046
			83	0.663898225871747	0.7108048151339528
			85	0.6484286249336046	0.7111275480057813
			87	0.6455734846865504	0.7096819730940409
			89	0.6475884332175817	0.7147406452026885
			91	0.6460630940574954	0.7034430255055726
			93	0.6427436328232249	0.7010815353840871
			95	0.6577728182558763	0.7092204809659383
			97	0.654836250185979	0.7008445031365073
			99	0.6360271080592259	0.7037307441270609
			101	0.6333690590030778	0.7030180427920791
			103	0.6318668249600476	0.6999007346676585
			105	0.6246765883561535	0.6990749378415549
			107	0.6253582971009171	0.6977632870443894
			109	0.6242740308514589	0.6956233607051137
			111	0.6238084539071662	0.6944569479008456
			113	0.6180224254500141	0.6955209468444596
			115	0.6171099569838884	0.6956002080821572
			117	0.6131144020250396	0.6936738979535368
			119	0.6088125797368547	0.6936733216676199
			Average	0.7130329631175655	0.7271343808421905

**No. of features**	**Metric**	**Measure**	**k**	**Reuters-8**	**Web-Kb**

ALL	BLAB_SM	g Measure	1	0.9084632242360435	0.8161419153827852
			3	0.8980865935012032	0.8147163870278432
			5	0.9067457901367432	0.8376926244756131
			7	0.8932264894915457	0.8451274108678803
			9	0.8886367298451194	0.8398400371966197
			11	0.8780742539489372	0.8367513524350162
			13	0.8721266582101068	0.8350866493970043
			15	0.8666685263322284	0.831622422395063
			17	0.8527751744845485	0.8348222157550209
			19	0.8591197729775433	0.8364772744147132
			21	0.8440449256402538	0.8346079951912366
			23	0.8451586053717836	0.8363950305815292
			25	0.8405288925674572	0.8307470856994592
			27	0.8354766023921866	0.8339883725461067
			29	0.8325403929596263	0.8308413545592886
			31	0.8326985981186216	0.8269356400128213
			33	0.8301148796855847	0.82621164496161
			35	0.8312057360401619	0.8248709840782751
			37	0.8252070764204708	0.8238168632933657
			39	0.824279383075627	0.8267106005668899
			41	0.8171005002421968	0.8218430470831178
			43	0.8215567247530494	0.8196212813718042
			45	0.8128854324204333	0.8175265300372698
			47	0.8111107786033047	0.819097196830832
			49	0.8088182797117037	0.8164739984884711
			51	0.8154726063016331	0.8161992746742175
			53	0.8120203416650191	0.8184235620369672
			55	0.8046410080057358	0.8193394344898687
			57	0.8039200685496041	0.8179310438177151
			59	0.8027890380261339	0.8207458373962118
			61	0.8038236929825349	0.8212787696075806
			63	0.7994920509802854	0.8181110793090195
			65	0.798548275382059	0.8176592843442376
			67	0.796246641820478	0.8127503781648264
			69	0.7931883486448019	0.8176525493725513
			71	0.7958260670997652	0.8159529448978202
			73	0.7895703803326957	0.8147025143185045
			75	0.7892658368623056	0.8151211563350296
			77	0.7842743113431018	0.8116488265382794
			79	0.7819100327915511	0.8128250301592806
			81	0.7752376049593269	0.8118937601205616
			83	0.7743575765376439	0.8111471243303127
			85	0.7689824610119123	0.811445370179028
			87	0.7664574210753902	0.8113070353684974
			89	0.76744268382795	0.8142513672760013
			91	0.7657844839730661	0.8084480080046127

			93	0.7645655407004347	0.8063357150246178
			95	0.7686010380256835	0.8092913597583294
			97	0.7669651087905733	0.8059294942008626
			99	0.7598234735683156	0.8069039361078126
			101	0.7579875119385479	0.8063178091187129
			103	0.7578098622106644	0.8050491540604933
			105	0.7543182339102542	0.8042712198990954
			107	0.7532097339812889	0.8030455972472381
			109	0.7517356915701682	0.8027024809939481
			111	0.7514314481860361	0.8016154338428169
			113	0.7477091560199821	0.802362942724085
			115	0.7471617226477212	0.8021559871161799
			117	0.7433686699726767	0.8009094708736411
			119	0.7412932189074695	0.8007263371571822
			Average	0.8065313560628213	0.8182402867252629

**No. of features**	**Metric**	**Measure**	**k**	**Reuters-8**	**Web-Kb**
ALL	BLAB_SM	Average Mean Precison	1	0.7523752085367694	0.6231205411228862
			3	0.7385754473788877	0.6430034343384782
			5	0.7621631663766981	0.6797113310599752
			7	0.7319350732624682	0.6974091514630626
			9	0.7291155580604117	0.6873466221288034
			11	0.7176203980586175	0.6825044391788027
			13	0.7005213563255008	0.6786838083885748
			15	0.6879547266636628	0.6749364373880243
			17	0.6628856097277198	0.6806419406254205
			19	0.676977307725456	0.6836945626951532
			21	0.6485569709896064	0.6806097502746645
			23	0.6526313829350257	0.684703636587195
			25	0.6438550899415241	0.6745589495698323
			27	0.6338842798819666	0.6784971298378363
			29	0.6283557065795828	0.6744080796710248
			31	0.6251197821996313	0.6670911608617718
			33	0.6267117025492719	0.6664476090049694
			35	0.6303490823896554	0.6640539034863684
			37	0.619855723995166	0.662056519881712
			39	0.6245683505994308	0.6661913602717325
			41	0.611531385082114	0.6587869280231166
			43	0.620108127849576	0.6546397928040959
			45	0.6058486328292461	0.6520773219698983
			47	0.602267211071071	0.6543465438542078
			49	0.5999559623945083	0.6501162721635666
			51	0.6113750551411441	0.6494822476824667

			53	0.6063393353770907	0.6537797570126822
			55	0.5936314065156953	0.6543867449150681
			57	0.5918239249651707	0.6527352526791238
			59	0.5901847950819725	0.6583794654618891
			61	0.5916227242030959	0.6590541536257175
			63	0.5841914858472957	0.6529960873978441
			65	0.5824857072771454	0.6525762192370049
			67	0.5797239629251568	0.6446783577383963
			69	0.5748038405432536	0.6531872471910393
			71	0.5816741501046879	0.6506467411580638
			73	0.5670938189386616	0.6493962557387877
			75	0.5677604793378817	0.6486249112873185
			77	0.5600942139656803	0.6420066384558187
			79	0.5565637473150342	0.6440455669976498
			81	0.5455002865013594	0.6431917340156583
			83	0.544811312730056	0.6417274920968427
			85	0.5369647206433745	0.6421152023448528
			87	0.5329882884766144	0.6419302414050219
			89	0.5361681282860318	0.6468796202535336
			91	0.5339444988905938	0.6373024741725013
			93	0.5293564805132261	0.6342302398963392
			95	0.5369457897532637	0.6394761884397713
			97	0.5357527322399598	0.633796766369581
			99	0.5230526040131435	0.6358482579414318
			101	0.5195190928316213	0.6347529070074893
			103	0.5174925890784823	0.632382243832982
			105	0.5099635822361801	0.6314224714760964
			107	0.5110566508599397	0.6296372895741498
			109	0.5096836691594337	0.6286856650082262
			111	0.5092986168305139	0.6281979713652234
			113	0.5017081253678877	0.6297072862520108
			115	0.5004942102792522	0.629789021914902
			117	0.49541647411846157	0.6272787271902506
			119	0.4928220792794072	0.6272528043653398
			Average	0.5949338637171891	0.6530202913025375

SMTP Similarity Measure

No. of features	Metric	Measure	k	Reuters-8	Web-Kb
**All**	**Smtp**	**Accuracy**	1	0.9242752055387278	0.7805071315372425
			3	0.9316313284292514	0.8177496038034865

			5	0.9337948939852877	0.8193343898573693
			7	0.9294677628732151	0.8225039619651348
			9	0.927736910428386	0.8264659270998416
			11	0.9281696235395932	0.8272583201267829
			13	0.9255733448723497	0.8232963549920761
			15	0.9208135006490696	0.8217115689381933
			17	0.9225443530938987	0.820919175911252
			19	0.9221116399826915	0.8248811410459588
			21	0.9212462137602769	0.820919175911252
			23	0.9208135006490696	0.8177496038034865
			25	0.9186499350930333	0.8169572107765452
			27	0.9182172219818261	0.8185419968304279
			29	0.9177845088706188	0.8177496038034865
			31	0.9186499350930333	0.8161648177496038
			33	0.916486369536997	0.8114104595879557
			35	0.9138900908697534	0.8090332805071315
			37	0.9134573777585461	0.8114104595879557
			39	0.9104283859800952	0.8114104595879557
			41	0.9082648204240589	0.8098256735340729
			43	0.9086975335352662	0.8082408874801902
			45	0.9082648204240589	0.8090332805071315
			47	0.906966681090437	0.8042789223454834
			49	0.9073993942016443	0.8026941362916006
			51	0.9056685417568152	0.805863708399366
			53	0.9043704024231934	0.8066561014263075
			55	0.9073993942016443	0.8019017432646592
			57	0.9073993942016443	0.8011093502377179
			59	0.9043704024231934	0.8003169572107766
			61	0.9039376893119861	0.7987321711568938
			63	0.9022068368671571	0.8003169572107766
			65	0.9000432713111207	0.8011093502377179
			67	0.898745131977499	0.8003169572107766
			69	0.8978797057550844	0.8003169572107766
			71	0.8970142795326699	0.8003169572107766
			73	0.8952834270878408	0.8003169572107766
			75	0.8944180008654262	0.7987321711568938
			77	0.8948507139766335	0.7995245641838352
			79	0.8948507139766335	0.7963549920760697
			81	0.8944180008654262	0.7963549920760697
			83	0.8931198615318044	0.7971473851030111
			85	0.8926871484205972	0.7963549920760697
			87	0.8905235828645608	0.7971473851030111
			89	0.8913890090869754	0.7931854199683043
			91	0.8922544353093899	0.7979397781299524
			93	0.8918217221981826	0.7955625990491284
			95	0.8913890090869754	0.7923930269413629
			97	0.8900908697533535	0.7939778129952456
			99	0.8900908697533535	0.7931854199683043
			101	0.889225443530939	0.7939778129952456

			103	0.889225443530939	0.794770206022187
			105	0.8887927304197317	0.7939778129952456
			107	0.8870618779749027	0.794770206022187
			109	0.88749459108611	0.7916006339144216
			111	0.8866291648636954	0.7916006339144216
			113	0.8857637386412809	0.7931854199683043
			115	0.8848983124188663	0.7908082408874801
			117	0.8853310255300736	0.7900158478605388
			119	0.8848983124188663	0.7900158478605388
			Average	0.9045146401269293	0.8043317485472785

No. of features	Metric	Measure	k	Reuters-8	Web-Kb
**All**	**Smtp**	**Precision**	1	0.8598636149164065	0.7733549612838566
			3	0.8936548453581211	0.8077991049285923
			5	0.8919109409563388	0.8305637028174071
			7	0.898538569890855	0.8415905248305592
			9	0.8992701559174086	0.848202588875691
			11	0.9021326913899743	0.8551418766957511
			13	0.9019175303978881	0.8574875108167825
			15	0.8982767934896341	0.8533762598777066
			17	0.9031778176533807	0.8526680010036477
			19	0.8992240161136819	0.8566328111896753
			21	0.8963996545048238	0.8506025383328469
			23	0.8930771588153926	0.8486243002220797
			25	0.894157317591062	0.8481989672897815
			27	0.8940870829679358	0.8464027991486713
			29	0.8963235886197198	0.8561594660278871
			31	0.9036206935066031	0.851003129853749
			33	0.8916001893112713	0.8510819989263076
			35	0.8907227384192096	0.8549835781402177
			37	0.8920673995837369	0.8524347864419048
			39	0.8894516845220165	0.8534729787677902
			41	0.8879753774375243	0.8517651133976778
			43	0.8882006424973032	0.8498797384535117
			45	0.8942570004799603	0.8562178218723188
			47	0.8926078015179228	0.8521712685784071
			49	0.8892938787912226	0.8493249447075304
			51	0.8920664624548895	0.8517264855138043
			53	0.8896602259052722	0.852994593439518
			55	0.8984228826950011	0.8496760493598225
			57	0.8970431495169732	0.8492748067297209
			59	0.8924767322530782	0.8551806643291019
			61	0.8911068619906188	0.8536525966104775
			63	0.8904229002403357	0.8483033388975519
			65	0.886612348873874	0.8488988096097871
			67	0.8871621726929569	0.8483432485361166
			69	0.8840646879557601	0.8548519047820615

			71	0.8845189045103138	0.8546699411414339
			73	0.8822832359671995	0.8550432436635103
			75	0.8893354813451544	0.8552875082502889
			77	0.8898930257661137	0.8554241424389022
			79	0.8890447258652208	0.8533380841152328
			81	0.9018008009719044	0.8530807353698227
			83	0.9004308927113689	0.853868528244595
			85	0.899075303636018	0.8527135246466127
			87	0.8955641685904021	0.8527688278306298
			89	0.8965918860762092	0.8500844963405683
			91	0.8988203128685887	0.8538434136489461
			93	0.897776578312005	0.8520889284376376
			95	0.8995099466704488	0.8587619387403319
			97	0.897483819856021	0.8596559357880869
			99	0.8972174773030521	0.851141826013278
			101	0.8992236221678375	0.85142302531442
			103	0.900812288495878	0.8604612825485063
			105	0.9014771391166034	0.8601583372622977
			107	0.9010162764226224	0.8605001698295986
			109	0.9009858700265458	0.8584691221448558
			111	0.8997315133334643	0.859099375730486
			113	0.8972066080465328	0.8596398744343151
			115	0.8953488642143426	0.8584972609561753
			117	0.8968440093751121	0.85773707821968
			119	0.898413523076952	0.8578354926035354
			Average	0.8943542313992343	0.850960589400034

No. of features	Metric	Measure	k	Reuters-8	Web-Kb
**All**	**Smtp**	**Recall**	1	0.8186085027991887	0.7326923603200535
			3	0.8240652275241114	0.7710860571031855
			5	0.8175545178984254	0.7620940644094595
			7	0.7930643724247868	0.7607346400675518
			9	0.786530828563565	0.7684890871463099
			11	0.7892585658727878	0.7632318660829905
			13	0.7862329597601236	0.7556255394336859
			15	0.7517659356025135	0.7519981505509206
			17	0.7583078936904253	0.7476381380937421
			19	0.7558731185000704	0.7484144940653981
			21	0.7447764014496421	0.7473002228951872
			23	0.7450814867741912	0.7419860889367027
			25	0.7463078202707889	0.741013898387355
			27	0.737658929163515	0.7418716847930604
			29	0.7308354329361937	0.7426117384457269
			31	0.7374183389678908	0.7401470774576288
			33	0.7295430353865815	0.7291117825364286
			35	0.7267365216562105	0.7306947179332491
			37	0.7255548431284393	0.7319031913531793

			39	0.7203304120481913	0.7306383779432427
			41	0.7183047368641882	0.7280975837534671
			43	0.7121228293170965	0.7258696143182037
			45	0.7115229477169478	0.7262964345341059
			47	0.6993713337325205	0.7176928268914108
			49	0.6999602266240882	0.7147161779617124
			51	0.7011574115171635	0.7183473041361268
			53	0.684360215491365	0.7199920409782321
			55	0.698871539891238	0.7136637082367862
			57	0.6996241585392848	0.7117864258157474
			59	0.689100558700544	0.7112793264242667
			61	0.6846497175336186	0.7079898527400561
			63	0.6718101044125626	0.7084879176075161
			65	0.6600529533912334	0.7097436964934318
			67	0.6574964704704567	0.7080989596513265
			69	0.6512821538481373	0.7070374554023794
			71	0.6504193766850791	0.7046057681238743
			73	0.6484185041190655	0.7042168101676848
			75	0.6469613078684723	0.7033297873440354
			77	0.6470744180975602	0.7025720733255796
			79	0.6448443793153806	0.7003111301807232
			81	0.6476055347139467	0.7003111301807232
			83	0.6413829282474309	0.7008182295722039
			85	0.636105490801561	0.699249625931776
			87	0.6301013091696446	0.6991644582060778
			89	0.6325584979014974	0.6948698202629267
			91	0.6315298673100083	0.699439012018625
			93	0.6313481812634967	0.6975287558879933
			95	0.6333687036583872	0.6936650841346454
			97	0.629239913830701	0.6949118284965403
			99	0.629556440196448	0.6936268131926806
			101	0.626609328896533	0.6943664581630948
			103	0.6268472202277728	0.6961165646248215
			105	0.62308180249311	0.6954530528560847
			107	0.6187561299771089	0.6958037398703094
			109	0.6176212971766138	0.6931538387692635
			111	0.6168009104805952	0.6922487468975724
			113	0.6099566923237834	0.6931868124788565
			115	0.6071930364432669	0.6896227849820987
			117	0.6065645167328888	0.690020777462309
			119	0.6070568142999168	0.6879780481399935
			Average	0.6884365850783059	0.7180825609028388

No. of features	Metric	Measure	k	Reuters-8	Web-Kb
**All**	**Smtp**	**F Measure**	1	0.8359119281044443	0.7434417215248428
			3	0.8496046286488044	0.7817050205199569
			5	0.8477528178733075	0.7783442674444401

			7	0.828919934282331	0.7781947008717819
			9	0.8220295768361227	0.7874600981069939
			11	0.8283205761137502	0.7830217954672261
			13	0.8263698230328697	0.7739936792872943
			15	0.7920020726110437	0.7706850592581738
			17	0.8024303749434332	0.7648064404443443
			19	0.7942729482005391	0.7662076054728477
			21	0.7871702676986136	0.7657642861364364
			23	0.7857934630091821	0.7603140274111014
			25	0.7916088578485214	0.7596067263879549
			27	0.7818058618790874	0.7604546747546752
			29	0.7724529148464228	0.7614632671401993
			31	0.7849804125295261	0.7582729032444867
			33	0.7760364272079829	0.7452838487326918
			35	0.7739756322456229	0.7491570642189683
			37	0.7735851037042121	0.7501607120746365
			39	0.7695961254274245	0.7481052672914812
			41	0.7710933025149271	0.7449439504613501
			43	0.7630532547198756	0.7420089647454423
			45	0.7648032558441982	0.7431295034797393
			47	0.7496844855404505	0.7322881592586101
			49	0.7488688980858083	0.7273638222181726
			51	0.7547794509061843	0.731518850914959
			53	0.7300987596345535	0.7340989486439353
			55	0.7494751972314506	0.7268762585172094
			57	0.7500145754069979	0.724557928362092
			59	0.739353452386349	0.7244729994023749
			61	0.7353163256501529	0.7195232413400636
			63	0.7179915846287996	0.7188710254591615
			65	0.7018587186868588	0.7209303328745921
			67	0.7002964242732708	0.7186247513023901
			69	0.6940801381272028	0.7169415483123545
			71	0.6930333785855805	0.7128614653218137
			73	0.6908665283451901	0.7127679396111588
			75	0.690956623517818	0.7136091930057487
			77	0.6912892947345641	0.7106730075621942
			79	0.6883639935746444	0.7085215927075385
			81	0.6928051180862347	0.708459115853064
			83	0.6864775384545059	0.7090865123641574
			85	0.680920247778827	0.7062970478628483
			87	0.6740197379315142	0.705020718016871
			89	0.6760483467852392	0.7000533298359941
			91	0.6744840851110931	0.7056538477131253
			93	0.6742098854883104	0.7037790353414878
			95	0.6799149548570302	0.6994981035502355
			97	0.674625847318419	0.7005698291241729
			99	0.6761259532622287	0.6997266634261314
			101	0.6728218602036471	0.7002559712876122
			103	0.6734175539598038	0.701592661482788

			105	0.6691524657327464	0.7010412115088761
			107	0.6666044376808946	0.7013114462492097
			109	0.6638680333321614	0.6989068857220383
			111	0.6648062801017174	0.6970648220480462
			113	0.6548190238574337	0.6979388951248592
			115	0.6518748397074512	0.6922732265033849
			117	0.6499141957945771	0.6937785061419897
			119	0.6505104598910495	0.6894205062991661
			Average	0.7326219709128835	0.7297125830791245

**No. of features**	**Metric**	**Measure**	**k**	**Reuters-8**	**Web-Kb**
**All**	**Smtp**	**g Measure**	1	0.898186154025304	0.8216826304287296
			3	0.9019971011631044	0.8490753975340383
			5	0.898449335992961	0.8437326895979814
			7	0.8844555690174024	0.8432643465713541
			9	0.8806433132685395	0.8481585632663431
			11	0.8822140656271235	0.8451566202578895
			13	0.8802255977158652	0.8404330139529803
			15	0.8602673735913351	0.8380196985791334
			17	0.8641336548567871	0.8354340696114823
			19	0.8627125959173432	0.8363610127605892
			21	0.8562700247136099	0.8350874778183279
			23	0.856481905112551	0.8315671778150667
			25	0.8569663601964518	0.8308529426354367
			27	0.8518954477418609	0.8315880862469448
			29	0.8479266667723888	0.8318456345557073
			31	0.8517815155102519	0.8302674215032041
			33	0.847103564942328	0.8231712452927252
			35	0.8452738107954259	0.8235391368031079
			37	0.8444875822788298	0.82468989477939
			39	0.841162841448418	0.8238658853448038
			41	0.8398033018272747	0.8222408700014485
			43	0.836219526615799	0.8207256958022505
			45	0.8357792236632111	0.8210111547704005
			47	0.8284943687092339	0.8153957795869452
			49	0.8288989945865117	0.8135036262473432
			51	0.8294486573192845	0.816092002411039
			53	0.8193183716911838	0.8170728618483611
			55	0.8281483395730815	0.8126659413358314
			57	0.8286171163905756	0.8114059753307609
			59	0.8221110406651807	0.810947330368288
			61	0.8194334672040262	0.8088570240785508
			63	0.8115224243472582	0.8093982002245865
			65	0.8042435063816471	0.8102814839654638
			67	0.802595232984736	0.8091759910077234
			69	0.7987523274056509	0.808549427106883
			71	0.7981335306996414	0.8071578227643672

			73	0.7967692207109237	0.8068874133316366
			75	0.7957613683676419	0.8059765134837052
			77	0.7958540039389981	0.8057244796097834
			79	0.7944950192252626	0.8039082541754062
			81	0.7960612018748704	0.8038915737679618
			83	0.7921286582029472	0.8042766974848097
			85	0.7888402501457001	0.8033295596031225
			87	0.7849305531396943	0.8034221036081838
			89	0.7865400587380179	0.8002927216352933
			91	0.7859586475077457	0.803626382614618
			93	0.785814600591119	0.8021754070728344
			95	0.7869969425820883	0.7993669861535323
			97	0.7843297023298165	0.8003673392170848
			99	0.7845353414186801	0.7994346675428133
			101	0.7826136995268942	0.8000255544174
			103	0.7827140760444969	0.8011783684972361
			105	0.7803206295858195	0.8006552729326283
			107	0.7774236558026055	0.8010220152824364
			109	0.7767540304469389	0.7989786548975388
			111	0.7761768271895784	0.7984498080943813
			113	0.7717877377553901	0.7992323564115873
			115	0.7699930754856195	0.796776712108643
			117	0.7696297497797705	0.7968894647627643
			119	0.7698904068782796	0.7957326147293763
			Average	0.8210078894670179	0.8155649175606708

**No. of features**	**Metric**	**Measure**	**k**	**Reuters-8**	**Web-Kb**
**All**	**Smtp**	**Avg. Mean Precison**	1	0.7179472739053987	0.6315100165074012
			3	0.7422517436069287	0.6737315970004674
			5	0.7388986802553508	0.677185100848743
			7	0.7185846277640895	0.6812222301062247
			9	0.7123070293366344	0.6901608497061913
			11	0.7178564746337572	0.6888771023921754
			13	0.7148716573365932	0.6815593088039815
			15	0.6792198169099218	0.6770844237710036
			17	0.6889572346877637	0.6728267917027244
			19	0.6838220976141433	0.6757135905974889
			21	0.6724861174265475	0.67259281988019
			23	0.670055160107875	0.6673180882942507
			25	0.6728318559143069	0.6663914942186941
			27	0.6643807198851956	0.6670623976399704
			29	0.6585688272338088	0.6705627111844851
			31	0.6702747123605943	0.6664918787163592
			33	0.6555130015277337	0.6557402846811262
			35	0.6523695241074488	0.6585648130393488
			37	0.652004133835677	0.6591541978450204
			39	0.6461641164417671	0.6590452480448956

			41	0.6436623831534499	0.655883214005808
			43	0.6376133740890042	0.6530454487218023
			45	0.6408479355055341	0.6551905705338631
			47	0.6278239908835181	0.6454910666070285
			49	0.6264483504270629	0.6413401733714637
			51	0.6289919431087119	0.6454598638530887
			53	0.6108299164292466	0.6475960662805047
			55	0.6305436002907541	0.6405120496265637
			57	0.6300582078583847	0.6387679911010641
			59	0.6180259632271696	0.6396822843025822
			61	0.613509892099631	0.6358897937395989
			63	0.6013041959362238	0.63560854318835
			65	0.5869474286047136	0.6368368300133246
			67	0.5849953906904282	0.6351957199741678
			69	0.5777276687943665	0.6351391816448999
			71	0.5768238546621826	0.6326098988766187
			73	0.5735448052233237	0.6324098850244764
			75	0.574506682220561	0.631968640575466
			77	0.5750250514716303	0.6313934487322208
			79	0.5726332202754891	0.6286505619377167
			81	0.5804350616631457	0.6283827469835254
			83	0.5736236737948006	0.6291999096111267
			85	0.5678343341932465	0.6269936244686372
			87	0.5600875767791602	0.6270200046811163
			89	0.5629211077166647	0.6221375675812959
			91	0.563378025343704	0.627855904357572
			93	0.5627235966415965	0.6253615428346945
			95	0.5662536505831464	0.6224553906910867
			97	0.5610634452219746	0.6238042401273801
			99	0.5611308950503038	0.6215986250856764
			101	0.5593135648303008	0.6222543552517515
			103	0.5605561446444611	0.6251337133933148
			105	0.5573428388532162	0.6243650581060389
			107	0.5536002259004782	0.6246106420588082
			109	0.552441543429421	0.6214719925678792
			111	0.5512003593928633	0.6209071407996329
			113	0.542789910729728	0.6218769968822937
			115	0.5387590656008214	0.6179365026249948
			117	0.539012097905504	0.6182077255058196
			119	0.5402844806634515	0.615966422302759
			Average	0.6169330043130152	0.6443167713834459

Results of Jaccard (these results led us to exclude Jaccard from any further consideration)

No. of features	Metric	Measure	k	Reuters-8	Web-Kb
ALL	Jaccard	Accuracy	1	0.32064041540458676	0.3969889064976228
			3	0.5114668974469927	0.22107765451664024
			5	0.5106014712245781	0.21949286846275753
			7	0.5101687581133708	0.21949286846275753
			9	0.5101687581133708	0.21949286846275753
			11	0.5101687581133708	0.22107765451664024
			13	0.5093033318909563	0.22107765451664024
			15	0.5093033318909563	0.22107765451664024
			17	0.5093033318909563	0.22107765451664024
			19	0.5093033318909563	0.22107765451664024
			21	0.5093033318909563	0.22107765451664024
			23	0.5093033318909563	0.22107765451664024
			25	0.5093033318909563	0.22107765451664024
			27	0.5093033318909563	0.22107765451664024
			29	0.5093033318909563	0.22107765451664024
			31	0.5093033318909563	0.22107765451664024
			33	0.5093033318909563	0.22107765451664024
			35	0.5093033318909563	0.22107765451664024
			37	0.5093033318909563	0.22107765451664024
			39	0.5093033318909563	0.22107765451664024
			41	0.5093033318909563	0.22107765451664024
			43	0.5093033318909563	0.22107765451664024
			45	0.5093033318909563	0.22107765451664024
			47	0.5093033318909563	0.22107765451664024
			49	0.5093033318909563	0.22107765451664024
			51	0.5093033318909563	0.22107765451664024
			53	0.5093033318909563	0.22107765451664024
			55	0.5093033318909563	0.22107765451664024
			57	0.5093033318909563	0.22107765451664024
			59	0.5093033318909563	0.22107765451664024
			61	0.5093033318909563	0.22107765451664024
			63	0.5093033318909563	0.22107765451664024
			65	0.5093033318909563	0.22107765451664024
			67	0.5093033318909563	0.22107765451664024
			69	0.5093033318909563	0.22107765451664024
			71	0.5093033318909563	0.22107765451664024
			73	0.5093033318909563	0.22107765451664024
			75	0.5093033318909563	0.22107765451664024
			77	0.5093033318909563	0.22107765451664024
			79	0.5093033318909563	0.22107765451664024
			81	0.5093033318909563	0.22107765451664024
			83	0.5093033318909563	0.22107765451664024
			85	0.5093033318909563	0.22107765451664024
			87	0.5093033318909563	0.22107765451664024

			89	0.5093033318909563	0.22107765451664024
			91	0.5093033318909563	0.22107765451664024
			93	0.5093033318909563	0.22107765451664024
			95	0.5093033318909563	0.22107765451664024
			97	0.5093033318909563	0.22107765451664024
			99	0.5093033318909563	0.22107765451664024
			101	0.5093033318909563	0.22107765451664024
			103	0.5093033318909563	0.22107765451664024
			105	0.5093033318909563	0.22107765451664024
			107	0.5093033318909563	0.22107765451664024
			109	0.5093033318909563	0.22107765451664024
			111	0.5093033318909563	0.22107765451664024
			113	0.5093033318909563	0.22107765451664024
			115	0.5093033318909563	0.22107765451664024
			117	0.5093033318909563	0.22107765451664024
			119	0.5093033318909563	0.22107765451664024
			Average	0.5062599163421312	0.22393026941362945

No. of features	Metric	Measure	k	Reuters-8	Web-Kb
**ALL**	**Jaccard**	**Precision**	1	0.7381543921916593	0.6733213429256595
			3	0.5013286334056399	0.3050476947535771
			5	0.15752329865626355	0.05496031746031746
			7	0.18871805976613254	0.05496031746031746
			9	0.18871805976613254	0.05496031746031746
			11	0.18871805976613254	0.05526941362916006
			13	0.06366291648636954	0.05526941362916006
			15	0.06366291648636954	0.05526941362916006
			17	0.06366291648636954	0.05526941362916006
			19	0.06366291648636954	0.05526941362916006
			21	0.06366291648636954	0.05526941362916006
			23	0.06366291648636954	0.05526941362916006
			25	0.06366291648636954	0.05526941362916006
			27	0.06366291648636954	0.05526941362916006
			29	0.06366291648636954	0.05526941362916006
			31	0.06366291648636954	0.05526941362916006
			33	0.06366291648636954	0.05526941362916006
			35	0.06366291648636954	0.05526941362916006
			37	0.06366291648636954	0.05526941362916006
			39	0.06366291648636954	0.05526941362916006
			41	0.06366291648636954	0.05526941362916006
			43	0.06366291648636954	0.05526941362916006
			45	0.06366291648636954	0.05526941362916006
			47	0.06366291648636954	0.05526941362916006
			49	0.06366291648636954	0.05526941362916006

			51	0.06366291648636954	0.05526941362916006
			53	0.06366291648636954	0.05526941362916006
			55	0.06366291648636954	0.05526941362916006
			57	0.06366291648636954	0.05526941362916006
			59	0.06366291648636954	0.05526941362916006
			61	0.06366291648636954	0.05526941362916006
			63	0.06366291648636954	0.05526941362916006
			65	0.06366291648636954	0.05526941362916006
			67	0.06366291648636954	0.05526941362916006
			69	0.06366291648636954	0.05526941362916006
			71	0.06366291648636954	0.05526941362916006
			73	0.06366291648636954	0.05526941362916006
			75	0.06366291648636954	0.05526941362916006
			77	0.06366291648636954	0.05526941362916006
			79	0.06366291648636954	0.05526941362916006
			81	0.06366291648636954	0.05526941362916006
			83	0.06366291648636954	0.05526941362916006
			85	0.06366291648636954	0.05526941362916006
			87	0.06366291648636954	0.05526941362916006
			89	0.06366291648636954	0.05526941362916006
			91	0.06366291648636954	0.05526941362916006
			93	0.06366291648636954	0.05526941362916006
			95	0.06366291648636954	0.05526941362916006
			97	0.06366291648636954	0.05526941362916006
			99	0.06366291648636954	0.05526941362916006
			101	0.06366291648636954	0.05526941362916006
			103	0.06366291648636954	0.05526941362916006
			105	0.06366291648636954	0.05526941362916006
			107	0.06366291648636954	0.05526941362916006
			109	0.06366291648636954	0.05526941362916006
			111	0.06366291648636954	0.05526941362916006
			113	0.06366291648636954	0.05526941362916006
			115	0.06366291648636954	0.05526941362916006
			117	0.06366291648636954	0.05526941362916006
			119	0.06366291648636954	0.05526941362916006
			Average	0.09001596656359843	0.06971779566106667

**No. of features**	**Metric**	**Measure**	**k**	**Reuters-8**	**Web-Kb**
**ALL**	**Jaccard**	**Recall**	1	0.17237140539926543	0.25768044672296236
			3	0.13333997029048483	0.24968717524548792
			5	0.12957317073170732	0.2482078853046595
			7	0.12804878048780488	0.2482078853046595
			9	0.12804878048780488	0.2482078853046595
			11	0.12804878048780488	0.25

			13	0.125	0.25
			15	0.125	0.25
			17	0.125	0.25
			19	0.125	0.25
			21	0.125	0.25
			23	0.125	0.25
			25	0.125	0.25
			27	0.125	0.25
			29	0.125	0.25
			31	0.125	0.25
			33	0.125	0.25
			35	0.125	0.25
			37	0.125	0.25
			39	0.125	0.25
			41	0.125	0.25
			43	0.125	0.25
			45	0.125	0.25
			47	0.125	0.25
			49	0.125	0.25
			51	0.125	0.25
			53	0.125	0.25
			55	0.125	0.25
			57	0.125	0.25
			59	0.125	0.25
			61	0.125	0.25
			63	0.125	0.25
			65	0.125	0.25
			67	0.125	0.25
			69	0.125	0.25
			71	0.125	0.25
			73	0.125	0.25
			75	0.125	0.25
			77	0.125	0.25
			79	0.125	0.25
			81	0.125	0.25
			83	0.125	0.25
			85	0.125	0.25
			87	0.125	0.25
			89	0.125	0.25
			91	0.125	0.25
			93	0.125	0.25
			95	0.125	0.25
			97	0.125	0.25
			99	0.125	0.25
			101	0.125	0.25
			103	0.125	0.25

			105	0.125	0.25
			107	0.125	0.25
			109	0.125	0.25
			111	0.125	0.25
			113	0.125	0.25
			115	0.125	0.25
			117	0.125	0.25
			119	0.125	0.25
			Average	0.12615718146474786	0.25003318796470714

**No. of features**	**Metric**	**Measure**	**k**	**Reuters-8**	**Web-Kb**
**ALL**	**Jaccard**	**F Measure**	1	0.14113715104000615	0.1571998407175876
			3	0.10083852892810236	0.09305178154540933
			5	0.0931784503244066	0.08999350227420402
			7	0.09036144578313252	0.08999350227420402
			9	0.09036144578313252	0.08999350227420402
			11	0.09036144578313252	0.09052563270603504
			13	0.08436066513761468	0.09052563270603504
			15	0.08436066513761468	0.09052563270603504
			17	0.08436066513761468	0.09052563270603504
			19	0.08436066513761468	0.09052563270603504
			21	0.08436066513761468	0.09052563270603504
			23	0.08436066513761468	0.09052563270603504
			25	0.08436066513761468	0.09052563270603504
			27	0.08436066513761468	0.09052563270603504
			29	0.08436066513761468	0.09052563270603504
			31	0.08436066513761468	0.09052563270603504
			33	0.08436066513761468	0.09052563270603504
			35	0.08436066513761468	0.09052563270603504
			37	0.08436066513761468	0.09052563270603504
			39	0.08436066513761468	0.09052563270603504
			41	0.08436066513761468	0.09052563270603504
			43	0.08436066513761468	0.09052563270603504
			45	0.08436066513761468	0.09052563270603504
			47	0.08436066513761468	0.09052563270603504
			49	0.08436066513761468	0.09052563270603504
			51	0.08436066513761468	0.09052563270603504
			53	0.08436066513761468	0.09052563270603504
			55	0.08436066513761468	0.09052563270603504
			57	0.08436066513761468	0.09052563270603504
			59	0.08436066513761468	0.09052563270603504
			61	0.08436066513761468	0.09052563270603504
			63	0.08436066513761468	0.09052563270603504
			65	0.08436066513761468	0.09052563270603504

			67	0.08436066513761468	0.09052563270603504
			69	0.08436066513761468	0.09052563270603504
			71	0.08436066513761468	0.09052563270603504
			73	0.08436066513761468	0.09052563270603504
			75	0.08436066513761468	0.09052563270603504
			77	0.08436066513761468	0.09052563270603504
			79	0.08436066513761468	0.09052563270603504
			81	0.08436066513761468	0.09052563270603504
			83	0.08436066513761468	0.09052563270603504
			85	0.08436066513761468	0.09052563270603504
			87	0.08436066513761468	0.09052563270603504
			89	0.08436066513761468	0.09052563270603504
			91	0.08436066513761468	0.09052563270603504
			93	0.08436066513761468	0.09052563270603504
			95	0.08436066513761468	0.09052563270603504
			97	0.08436066513761468	0.09052563270603504
			99	0.08436066513761468	0.09052563270603504
			101	0.08436066513761468	0.09052563270603504
			103	0.08436066513761468	0.09052563270603504
			105	0.08436066513761468	0.09052563270603504
			107	0.08436066513761468	0.09052563270603504
			109	0.08436066513761468	0.09052563270603504
			111	0.08436066513761468	0.09052563270603504
			113	0.08436066513761468	0.09052563270603504
			115	0.08436066513761468	0.09052563270603504
			117	0.08436066513761468	0.09052563270603504
			119	0.08436066513761468	0.09052563270603504
			Average	0.08602857308455167	0.09165236546529218

**No. of features**	**Metric**	**Measure**	**k**	**Reuters-8**	**Web-Kb**
**ALL**	**Jaccard**	**g Measure**	1	0.38928521652432113	0.4404205864122005
			3	0.34169159656632886	0.432700865147008
			5	0.33678834464593904	0.4312708318887152
			7	0.33476993947454486	0.4312708318887152
			9	0.33476993947454486	0.4312708318887152
			11	0.33476993947454486	0.4330127018922193
			13	0.33071891388307384	0.4330127018922193
			15	0.33071891388307384	0.4330127018922193
			17	0.33071891388307384	0.4330127018922193
			19	0.33071891388307384	0.4330127018922193
			21	0.33071891388307384	0.4330127018922193
			23	0.33071891388307384	0.4330127018922193
			25	0.33071891388307384	0.4330127018922193
			27	0.33071891388307384	0.4330127018922193

			29	0.33071891388307384	0.4330127018922193
			31	0.33071891388307384	0.4330127018922193
			33	0.33071891388307384	0.4330127018922193
			35	0.33071891388307384	0.4330127018922193
			37	0.33071891388307384	0.4330127018922193
			39	0.33071891388307384	0.4330127018922193
			41	0.33071891388307384	0.4330127018922193
			43	0.33071891388307384	0.4330127018922193
			45	0.33071891388307384	0.4330127018922193
			47	0.33071891388307384	0.4330127018922193
			49	0.33071891388307384	0.4330127018922193
			51	0.33071891388307384	0.4330127018922193
			53	0.33071891388307384	0.4330127018922193
			55	0.33071891388307384	0.4330127018922193
			57	0.33071891388307384	0.4330127018922193
			59	0.33071891388307384	0.4330127018922193
			61	0.33071891388307384	0.4330127018922193
			63	0.33071891388307384	0.4330127018922193
			65	0.33071891388307384	0.4330127018922193
			67	0.33071891388307384	0.4330127018922193
			69	0.33071891388307384	0.4330127018922193
			71	0.33071891388307384	0.4330127018922193
			73	0.33071891388307384	0.4330127018922193
			75	0.33071891388307384	0.4330127018922193
			77	0.33071891388307384	0.4330127018922193
			79	0.33071891388307384	0.4330127018922193
			81	0.33071891388307384	0.4330127018922193
			83	0.33071891388307384	0.4330127018922193
			85	0.33071891388307384	0.4330127018922193
			87	0.33071891388307384	0.4330127018922193
			89	0.33071891388307384	0.4330127018922193
			91	0.33071891388307384	0.4330127018922193
			93	0.33071891388307384	0.4330127018922193
			95	0.33071891388307384	0.4330127018922193
			97	0.33071891388307384	0.4330127018922193
			99	0.33071891388307384	0.4330127018922193
			101	0.33071891388307384	0.4330127018922193
			103	0.33071891388307384	0.4330127018922193
			105	0.33071891388307384	0.4330127018922193
			107	0.33071891388307384	0.4330127018922193
			109	0.33071891388307384	0.4330127018922193
			111	0.33071891388307384	0.4330127018922193
			113	0.33071891388307384	0.4330127018922193
			115	0.33071891388307384	0.4330127018922193
			117	0.33071891388307384	0.4330127018922193
			119	0.33071891388307384	0.4330127018922193

			Average	0.3321816054307702	0.4330438758549573

No. of features	Metric	Measure	k	Reuters-8	Web-Kb
ALL	Jaccard	Avg. Mean Precison	1	0.16555048602727107	0.25564814324564417
			3	0.132563198504498	0.25086296393439544
			5	0.12837799280197176	0.2496931195743032
			7	0.12799574548976606	0.2496931195743032
			9	0.12799574548976606	0.2496931195743032
			11	0.12799574548976606	0.24999999999999997
			13	0.125	0.24999999999999997
			15	0.125	0.24999999999999997
			17	0.125	0.24999999999999997
			19	0.125	0.24999999999999997
			21	0.125	0.24999999999999997
			23	0.125	0.24999999999999997
			25	0.125	0.24999999999999997
			27	0.125	0.24999999999999997
			29	0.125	0.24999999999999997
			31	0.125	0.24999999999999997
			33	0.125	0.24999999999999997
			35	0.125	0.24999999999999997
			37	0.125	0.24999999999999997
			39	0.125	0.24999999999999997
			41	0.125	0.24999999999999997
			43	0.125	0.24999999999999997
			45	0.125	0.24999999999999997
			47	0.125	0.24999999999999997
			49	0.125	0.24999999999999997
			51	0.125	0.24999999999999997
			53	0.125	0.24999999999999997
			55	0.125	0.24999999999999997
			57	0.125	0.24999999999999997
			59	0.125	0.24999999999999997
			61	0.125	0.24999999999999997
			63	0.125	0.24999999999999997
			65	0.125	0.24999999999999997
			67	0.125	0.24999999999999997
			69	0.125	0.24999999999999997
			71	0.125	0.24999999999999997
			73	0.125	0.24999999999999997
			75	0.125	0.24999999999999997
			77	0.125	0.24999999999999997
			79	0.125	0.24999999999999997
			81	0.125	0.24999999999999997

			83	0.125	0.24999999999999997
			85	0.125	0.24999999999999997
			87	0.125	0.24999999999999997
			89	0.125	0.24999999999999997
			91	0.125	0.24999999999999997
			93	0.125	0.24999999999999997
			95	0.125	0.24999999999999997
			97	0.125	0.24999999999999997
			99	0.125	0.24999999999999997
			101	0.125	0.24999999999999997
			103	0.125	0.24999999999999997
			105	0.125	0.24999999999999997
			107	0.125	0.24999999999999997
			109	0.125	0.24999999999999997
			111	0.125	0.24999999999999997
			113	0.125	0.24999999999999997
			115	0.125	0.24999999999999997
			117	0.125	0.24999999999999997
			119	0.125	0.24999999999999997
			Average	0.1260079818967173	0.2500931744317158
